# IGF-IR determines the fates of BCR/ABL leukemia

**DOI:** 10.1186/s13045-015-0106-8

**Published:** 2015-02-04

**Authors:** Jingjing Xie, Xiaoli Chen, Junke Zheng, Chunling Li, Satomi Stacy, Martin Holzenberger, Xuemei Hu, Cheng Cheng Zhang

**Affiliations:** Taishan Scholar Immunology Program, Binzhou Medical University, 264003 Yantai, Shandong China; Departments of Physiology and Developmental Biology, University of Texas Southwestern Medical Center, 75390 Dallas, TX USA; INSERM and Sorbonne Universities, UPMC, Research Center UMR938, 75012 Paris, France

**Keywords:** Leukemia, Chronic myeloid leukemia, Acute lymphoblastic leukemia, Hematopoietic stem cells, Tyrosine kinase receptor, BCR/ABL, IGF-IR

## Abstract

**Background:**

The tyrosine kinase receptor insulin-like growth factor 1 receptor (IGF-IR) contributes to the initiation and progression of many types of malignancies. We previously showed that IGF-2, which binds IGF-IR, is an extrinsic factor that supports the *ex vivo* expansion of hematopoietic stem cells (HSCs). We also demonstrated that IGF-IR is not required for HSC activity *in vivo*.

**Methods and results:**

Here we investigated the role of IGF-IR in chronic myeloid leukemia (CML) using the retroviral BCR/ABL transplantation mouse model. Existing antibodies against IGF-IR are not suitable for flow cytometry; therefore, we generated a fusion of the human IgG Fc fragment with mutant IGF-2 that can bind to IGF-IR. We used this fusion protein to evaluate mouse primary hematopoietic populations. Through transplantation assays with IGF-IR^+^ and IGF-IR^−^ cells, we demonstrated that IGF-IR is expressed on all mouse HSCs. The expression of IGF-IR is much higher on CML cells than on acute lymphoblastic leukemia (ALL) cells. The depletion of IGF-IR expression in BCR/ABL^+^ cells led to the development of ALL (mostly T cell ALL) but not CML. Lack of IGF-IR resulted in decreased self-renewal of the BCR/ABL^+^ CML cells in the serial replating assay.

**Conclusion:**

IGF-IR regulates the cell fate determination of BCR/ABL^+^ leukemia cells and supports the self-renewal of CML cells.

## Introduction

Chronic myeloid leukemia (CML) is a hematopoietic malignancy that results from the t(9;22)(q34;q11) Philadelphia (Ph) chromosome translocation, which produces the BCR/ABL fusion oncogene [1-3]. Tyrosine kinase inhibitors (TKIs) effectively treat symptoms of CML patients, but the disease is not curable in most cases. Both BCR/ABL dependent and independent mechanisms contribute to TKI resistance. The BCR/ABL dependent resistance may result from duplication, overamplification, or mutation of the gene encoding BCR/ABL [4-7]. BCR/ABL independent resistance mechanisms result from drug efflux or influx or activation of alternative signaling pathways [8,9]. CML-initiating cells (CML-ICs) in patients have been shown to be insensitive to TKIs [10]. Novel targets in CML-ICs must be identified in order to develop therapeutic strategies to eliminate CML.

Insulin-like growth factor 1 receptor (IGF-IR) is a receptor tyrosine kinase. IGF-IR is a homodimer of α and β subunits that recognizes ligands IGF-1 and IGF-2 [11]. IGF-IR-mediated signaling is important for normal development and contributes to the initiation and progression of many types of solid malignancies [11-14]. IGF-IR also contributes to development of hematopoietic malignancies including acute myeloid leukemia (AML), T cell acute lymphoblastic leukemia (T-ALL), multiple myeloma, and CML. Autocrine IGF signaling, mediated through IGF-IR, supports the growth and survival of AML cells via the phosphoinositide 3-kinase/Akt pathway [15]. IGF-IR is also critical for the transformation of MLL-AF9 AML but is not required for AML propagation [16]. IGF-IR expression is essential for maintenance of T-ALL stem cells and is supported by Notch signaling [17,18]. Inhibition of IGF-IR signaling has a significant antitumor effect on multiple myeloma in a xenograft model [19]. BCR/ABL upregulates IGF-1 expression and IGF-1 is overexpressed in human CML samples. Aberrant autocrine IGF signaling supports progression to the CML blast crisis phase [20].

We previously reported that IGF signaling supports hematopoietic stem cell (HSC) expansion [21]. However, IGF-IR is dispensable for HSC activity in mice [22]. Here we sought to test whether a signaling that is dispensable to normal HSCs is important to the activity of malignant CML cells. We found that IGF-IR is highly expressed on HSCs, and levels of IGF-IR were much higher on CML cells than on ALL cells. Although IGF-IR is dispensable for activity of normal HSCs, it is critical to BCR/ABL leukemia fate determination and self-renewal of CML cells. Loss of IGF-IR in Ph^+^ cells resulted in development of ALL.

## Results

### IGF-IR is expressed on the surface of HSCs

**Figure 1:**
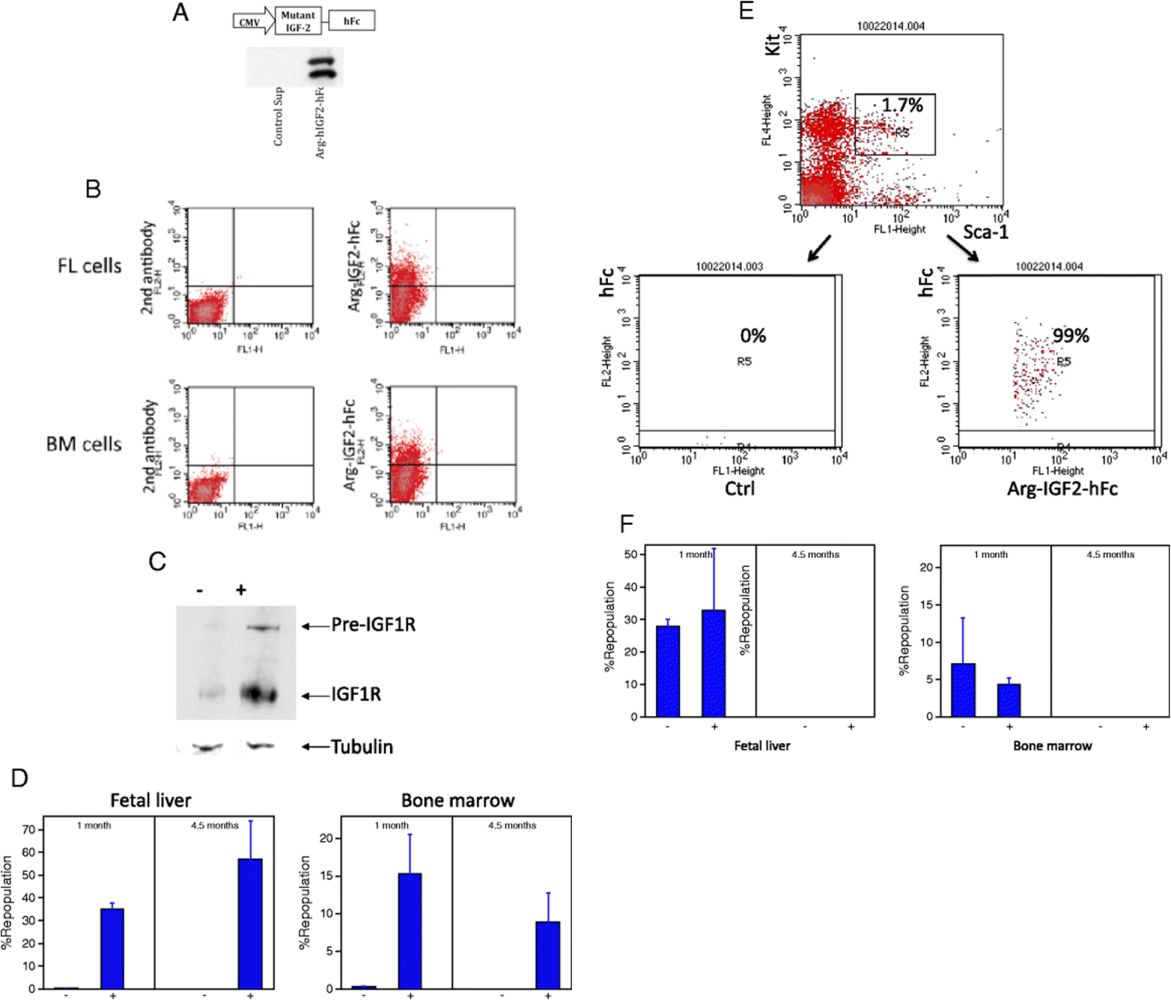
**IGF-2 receptor expression in fetal liver cells. (A)** Production and secretion of IGF2-hFc in transfected 293T cells. The upper panel shows a schematic of the plasmid expressing the human prepro-IGF-2 protein fused to a human IgG1 Fc fragment. The bottom panels show western Blots of conditioned medium collected 48 hours after transfection from 293T cells transfected with either control or Arg-IGF2-hFc vectors; blots were probed with antibodies against human IGF-2 (left) or human IgG1 (right). **(B)** Mouse fetal liver cells (FL) and adult bone marrow cells (BM) contain IGF-IR^+^ cells that bind to Arg-IGF-2-hFc as determined by flow cytometry. **(C)** Western blot of total fetal liver cells sorted based on binding of Arg-IGF2-hFc. Antibody against IGF-IR (Cell Signaling) was used to detect the expression of IGF-IR in flow cytometry-sorted Arg-IGF2-hFc^+^ and Arg-IGF2-hFc^−^ fetal liver cells. These results confirmed the specificity of Arg-IGF2-hFc binding to IGF-IR^+^ cells. Blotting with anti-beta-tubulin (Sigma) served as the loading control. **(D)** All repopulating HSCs in the total population of bone marrow (BM) or fetal liver cells (FL) bind Arg-IGF2-hFc. One thousand CD45.2 bone marrow or fetal liver Arg-IGF2-hFc^+^ cells and 1,000 Arg-IGF2-hFc^−^ cells were transplanted together with 2 × 10^5^ CD45.1 competitor cells into lethally irradiated CD45.1 mice (n = 5). Peripheral blood cells were analyzed for the presence of CD45.2^+^ cells at 6 months after transplant. **(E)** All the mouse bone marrow Lin^−^ Sca1^+^ Kit^+^ cells are IGF-IR^+^ cells as determined by flow cytometry. Bone marrow Lin^−^ cells were gated. **(F)** All repopulating HSCs in the Lin^−^Sca-1^+^Kit^+^ cells bind Arg-IGF2-hFc. Fifty CD45.2 bone marrow or fetal liver Lin^−^Sca-1^+^Arg-IGF2-hFc^+^ and 100 Lin^−^Sca-1^+^IGF2-hFc^−^ cells were transplanted together with 2 × 10^5^ CD45.1 competitor cells into lethally irradiated CD45.1 mice (n = 4-5). Peripheral blood cells were analyzed for the presence of CD45.2^+^ cells at 6 months after transplant.

We previously showed that IGF-2 stimulates *ex vivo* expansion of HSCs [21]. There are three known receptors for IGF-2: IGF type 1 receptor (IGF-IR), IGF type 2 receptor (IGF-IIR), and insulin receptor. Using a fusion of IGF-2 and IgG Fc as an affinity reagent, we previously showed that all repopulating HSCs bound IGFs [21]. Because existing antibodies against IGF-IR are not suitable for flow cytometry, we were unable to determine whether IGF-IR was expressed on HSCs. Thus we generated a fusion protein of IgG Fc with a mutant IGF-2 that can bind only to IGF-IR but not IGF-IIR (Figure 1A). As described previously [23], Ala and Leu replacements of Arg54 and Arg55, respectively, led to an IGF2 (Arg-IGF2) incapable of binding to IGF-IIR. We used FACS to sort cells based on their abilities to bind to this mutant IGF2 (Figure 1B). The lysates of these sorted cells were analyzed by western blot with antibodies against IGF-IR, confirming that Arg-IGF2 specifically binds to IGF-IR (Figure 1C). About 30 ± 6% or 18 ± 5% of total fetal liver or bone marrow cells in mice were IGF-IR^+^ respectively (Figure 1B). By using sorted IGF-IR^+^ and IGF-IR^−^ cells in comparative long-term bone marrow repopulation assays, we demonstrated that all mouse fetal liver and adult bone marrow HSCs express IGF-IR (Figure 1D). Moreover, we used flow cytometry to show that, all Lin^−^Sca-1^+^Kit^+^ cells are IGF-IR^+^ (Figure 1E). Although approximately 50% of Lin^−^Sca-1^+^ cells are IGF-IR^+^ (not shown), all of the repopulating activity of Lin^−^Sca-1^+^ cells resided in the fetal liver and bone marrow Lin^−^Sca-1^+^IGF-hFc^+^ fraction (Figure 1F). That mouse HSCs express IGF-IR suggests a role of IGF signaling in physiology and pathogenesis of HSCs.

### IGF-IR regulates BCR/ABL leukemia fates

**Figure 2:**
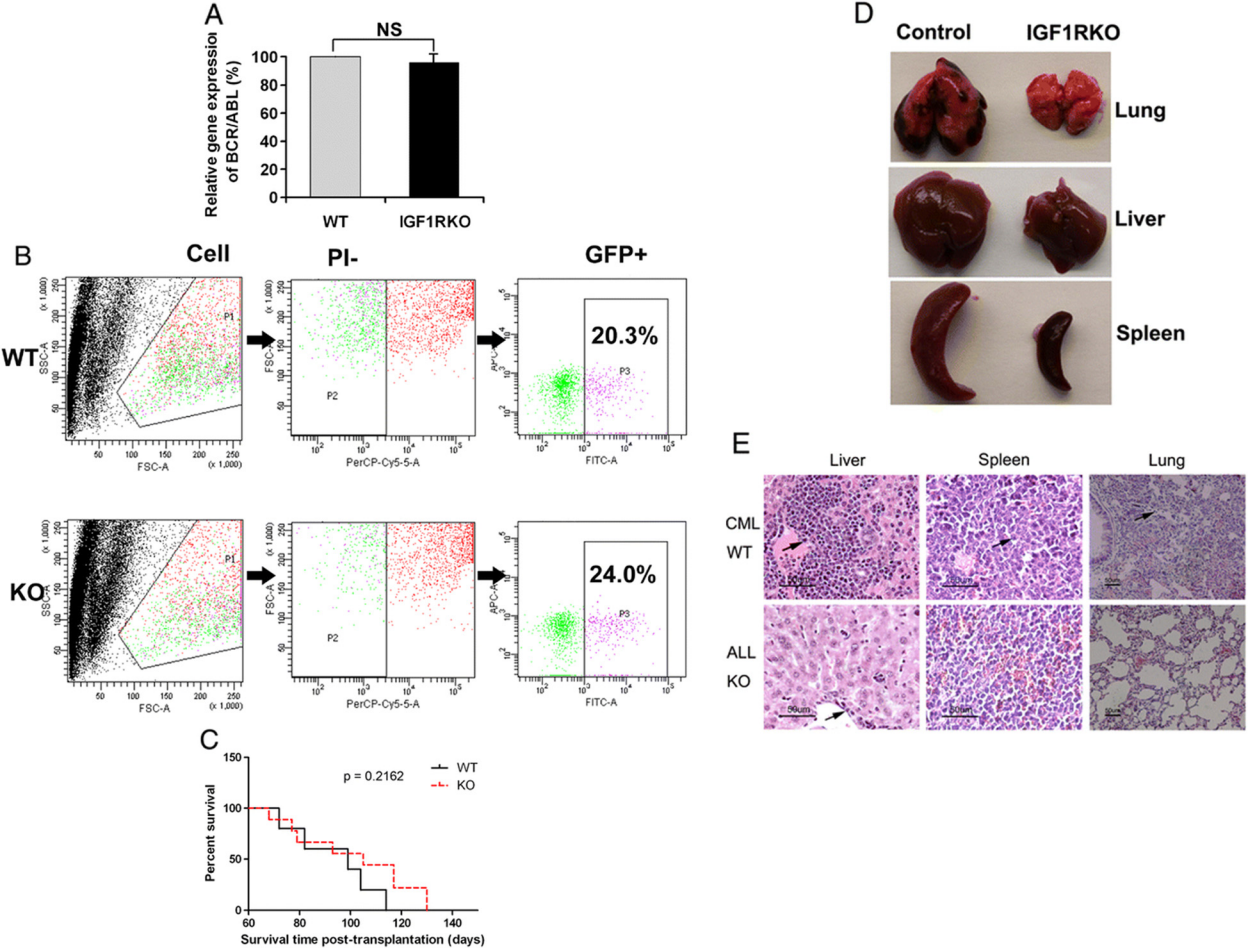
**Lack of IGF-IR alters leukemia development. (A)** Expression of mRNA encoding BCR/ABL in GFP^+^ WT and *IGF-IR*-null leukemia cells as determined by real-time RT-PCR. **(B)** Representative flow cytometry plots showing that WT and *IGF-IR*-null BCR/ABL transplanted mice have comparable GFP^+^ cells at 3 months after transplantation. **(C)** Survival curve of mice receiving BCR/ABL-infected WT or *IGF-IR*-null hematopoietic progenitors (n = 15). **(D)** Comparison of the sizes of lungs, livers, and spleens of representative mice transplanted with WT BCR/ABL cells and *IGF-IR*-null BCR/ABL cells at 3 months after transplantation. **(E)** Histological analysis of leukemia infiltration in the internal organs of WT and *IGF-IR*-null BCR/ABL cells transplanted mice at 3 months (hematoxylin/eosin staining).

The fact that IGF-IR is expressed on HSCs but does not play an essential role in regulation of HSC repopulation *in vivo* led us to investigate the role of IGF-IR-mediated signaling in hematopoietic malignancies. IGF-IR supports hematopoietic malignancies including AML, T-ALL, and multiple myeloma [15-19]. The presence of the BCR/ABL fusion is also correlated with elevated IGF-1 expression in human CML samples. Autocrine IGF signaling supports progression of the CML blast crisis phase, and, conversely, inhibition of IGF-1R reduces viability and proliferation of BCR-ABL^+^ cells [20]. We therefore used a retroviral BCR-ABL transplantation mouse model [24-26] to further study the role of IGF-IR in regulation of BCR/ABL leukemia development. Wild-type (WT) or *IGF-IR*-null donor fetal liver Lin^−^ cells infected by retroviral BCR-ABL-IRES-GFP were used to induce leukemia. The lack of IGF-IR did not change the level of expression of the mRNA encoding BCR/ABL (Figure 2A). We next investigated whether IGF-IR was required for the induction of leukemia by BCR/ABL. Mice transplanted with BCR/ABL-transduced WT and IGF-IR cells had similar GFP^+^ cells at 3 months (Figure 2B), suggesting IGF-IR is not essential for BCR/ABL leukemia propagation. Consistently, IGF-IR deficiency did not cause a significant alteration in the survival of leukemic mice (Figure 2C). However, there was a much less severe infiltration of *IGF-IR*-null myeloid leukemia cells into the lung, liver, and spleen compared to levels of WT cells (Figure 2D-E), suggesting that IGF-IR does impact leukemia development.

**Figure 3:**
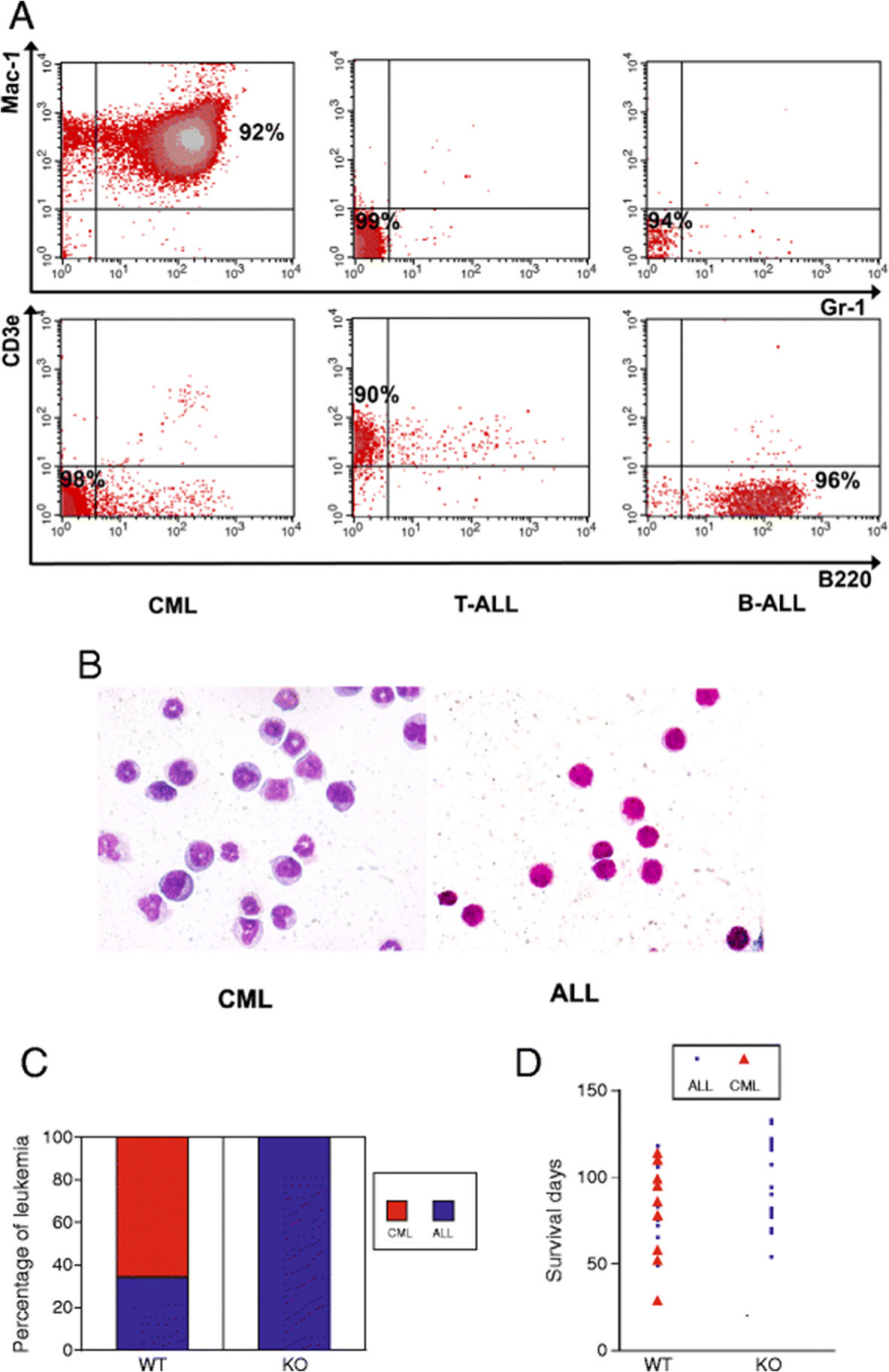
**IGF-IR deficiency alters BCR/ABL leukemia fates. (A)** Representative flow cytometry plots showing CML, T-ALL, and B-ALL cells in mice transplanted with WT or *IGF-IR*-null BCR/ABL cells. **(B)** Cytospin staining of CML and ALL in mice transplanted with WT or *IGF-IR*-null BCR/ABL cells. **(C)** Percentages of different types of leukemia developed by mice transplanted with BCR/ABL-infected WT or *IGF-IR*-null hematopoietic progenitors. **(D)** Survival curves of mice receiving BCR/ABL-infected WT or *IGF-IR*-null hematopoietic progenitors that developed different types of leukemia (n = 15).

Samples from mice transplanted with *IGF-IR*-null BCR/ABL cells and those transplanted with WT BCR/ABL cells were analyzed by flow cytometry and cytospin staining for cells characteristic of CML and ALL (Figure 3A-B). Over 60% of WT BCR/ABL-infected cells developed into CML, and the rest developed into ALL. In contrast, all *IGF-IR*-null BCR/ABL cells resulted in ALL, with T-ALL observed in most cases (Figure 3C-D, 26 out of 28 cases were T-ALL). While the mice with the null leukemia might show a delayed trend of death, the difference in types of leukemia did not significantly alter the survival of mice infected with WT and *IGF-IR*-null BCR/ABL cells (Figure 3D).

In addition to infecting fetal liver Lin^−^ cells, we also followed the published protocols [25,26] to infect 5-FU treated bone marrow cells with BCR/ABL-IRES-GFP retrovirus. Concordant with reported results, all the recipient mice transplanted with BCR/ABL virus infected WT cells developed CML-like disease. Three recipient mice survived 20, 29, and 31 days respectively. In contrast, all the recipient mice transplanted with *IGF-IR*-null BCR/ABL cells developed ALL and survived 29, 35, and 46 days respectively. These results confirmed that the deficiency of IGF-IR results in alteration of fates of BCR/ABL induced leukemia.

### Deficiency in IGF-IR decreases self-renewal of CML cells

**Figure 4:**
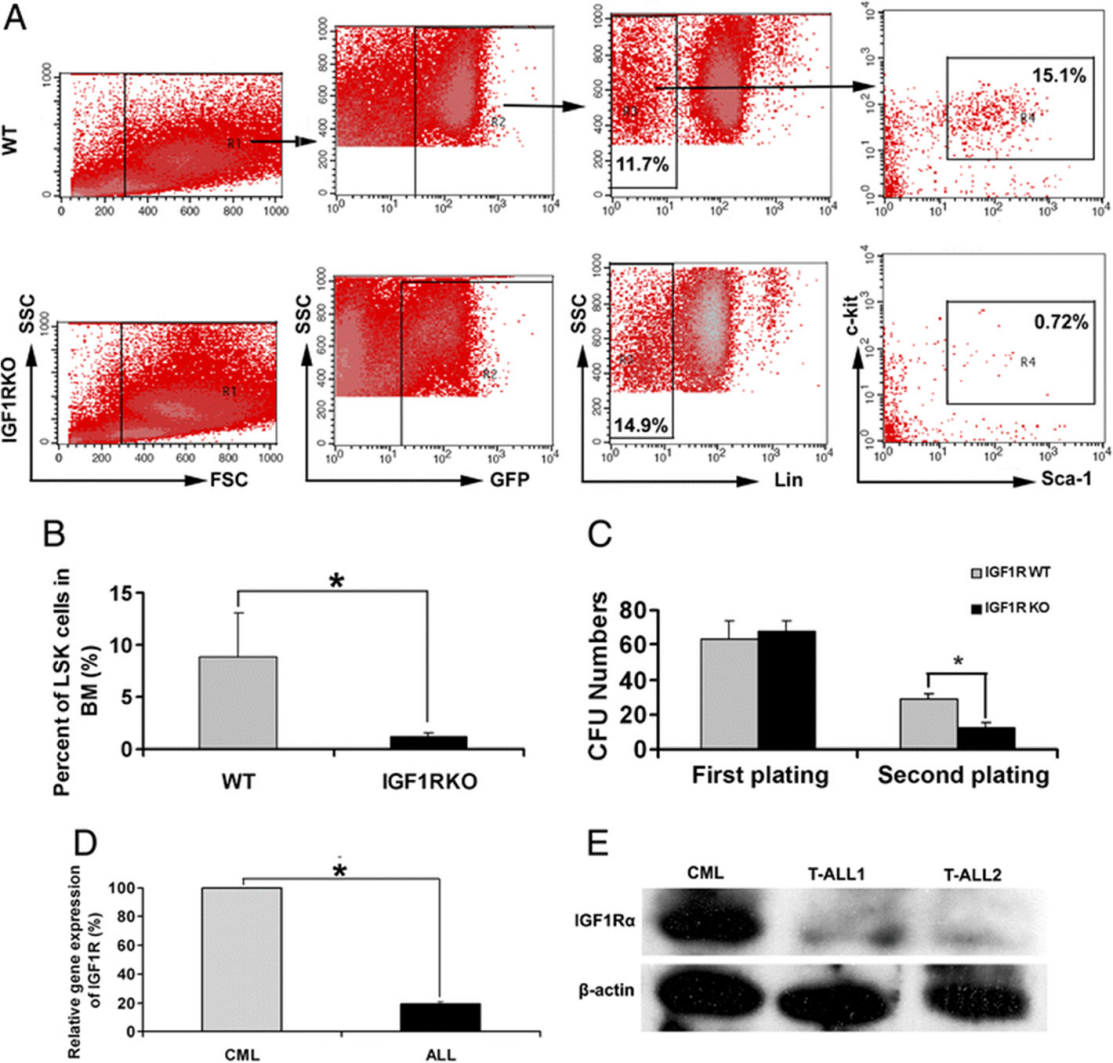
**Lack of IGF-IR decreases self-renewal of CML cells.** **(A-B)** LSK percentages dramatically decreased in leukemia bone marrow from mice transplanted with *IGF-IR*-null compared to WT BCR/ABL cells as shown in **(A)** representative flow cytometry plots and **(B)** as summarized in the bar graph. **(C)** *IGF-IR*-null leukemia cells have dramatically decreased CFU forming ability in second replating (n = 3). **(D-E)** Expression of IGF-IR in WT and *IGF-IR*-null BCR/ABL leukemia cells as determined by **(D)** real-time RT-PCR and **(E)** western blotting.

We next assessed whether IGF-IR regulates self-renewal of BCR/ABL leukemia cells. IGF-IR deficiency led to dramatically decreased LSK percentages in leukemia bone marrow (Figure 4A-B). Colony formation assays showed that WT and *IGF-IR*-null BCR/ABL cells had similar colony forming abilities in primary plating; however, *IGF-IR*-null cells had decreased colony forming capacity upon replating (Figure 4C). These results suggest that IGF-IR supports the self-renewal of CML cells. IGF-IR was expressed at significantly higher levels by BCR/ABL^+^ CML cells than by T-ALL or B-ALL cells (Figure 4D-E). Together, our results suggest that the lack of IGF-IR on CML cells shifts BCR/ABL leukemia cell fate from CML to ALL.

## Discussion

Previously, we showed that IGF-2 stimulates the *ex vivo* expansion of HSCs [21]. IGF-IR is not required for *in vivo* repopulation of HSCs, however [22]. Here, we demonstrated that IGF-IR is highly expressed on HSCs and CML cells but is only expressed at low levels on ALL cells. Although IGF-IR is dispensable for normal HSC maintenance, it is critical to BCR/ABL leukemia fate determination. Loss of IGF-IR in Ph^+^ leukemia resulted in development of ALL. That IGF-IR supports CML cell self-renewal is concordant with the reported function of IGF-1R in self-renewal of embryonic stem cells [27] and in *ex vivo* expansion of HSCs [21]. Like other signaling pathways, including Wnt/β-catenin and Hedgehog [28], the IGF-IR signaling system appears to be dispensable in normal HSCs but activated in leukemia cells. Such signaling pathways contribute specifically to cancer initiation or progression.

An important question is whether IGF-IR plays different roles in different types of cells. Our and others’ studies clearly indicated that IGF-IR has distinct functions in different contexts. It is well documented that IGF-IR is important in a variety of functions in different cancer types including proliferation, adhesion, migration, survival, and metastases [12]. While it is not required for *in vivo* HSC repopulation, IGF-IR is required for various leukemia development. As shown here, IGF-IR is necessary for fate determination and self-renewal of CML cells and blocking IGF-IR signaling inhibits CML development but leads to Ph^+^ ALL. Consistently, IGF-IR transforms MLL-AF9 AML progenitors but does not to stimulate leukemia propagation, and blocking IGF-IR signaling inhibits AML development [16]. IGF-IR has different roles in different types of T-ALL. While high levels of IGF-IR support the activity of T-LBL stem cells [17], it is clear from our study that BCR/ABL driven T-ALL does not express a significant level of IGF-IR and IGF-IR is not required for Ph^+^ T-ALL development.

This study raises provocative questions regarding extrinsic signaling for leukemia stem cells. Is IGF-IR signaling in CML cells independent of BCR/ABL-induced signaling? How does IGF signaling, together with other extrinsic and intrinsic pathways (IL-6, Wnt, Hedgehog, BMPs, selectin, TGF-β, Alox5, CD25, among others) [9,28-33], regulate the fates of CML cells including self-renewal, differentiation, apoptosis, and migration? Given extensive aberrant signaling in CML cells, we expect that the combined use of TKIs and inhibition of a limited number of key extrinsic pathways, including IGF-IR signaling, will lead to the development of novel strategies for treating CML.

## Conclusion

We developed a novel method to mark IGF-IR on the surface of cells. Using this flow cytometry-based method and reconstitution analyses, we found that IGF-IR is expressed on all mouse HSCs. The expression of IGF-IR was dramatically higher on CML cells than on ALL cells. The knockout of *IGF-IR* in BCR/ABL^+^ cells led to the development of ALL (mostly T-ALL) but not CML. Lack of IGF-IR resulted in decreased self-renewal of the BCR/ABL^+^ CML cells. Therefore, IGF-IR directs BCR/ABL^+^ leukemia cells toward the myeloid fate. Because IGF-IR is dispensable for the activity of HSCs but regulates BCR/ABL leukemia cell fates and supports self-renewal of CML cells, targeting IGF-IR may be an ideal anti-leukemia strategy. Inhibition of the IGF-IR axis may contribute to the development of future combination therapies that selectively target multiple key signaling pathways unique to CML cells.

## Methods

### Animals

C57 BL/6 CD45.2 and CD45.1 mice were purchased from Jackson Lab or National Cancer Institute. IGF-IR^+/-^ mice as previously described [34] were in a pure C57BL/6 background. Mice were maintained at the University of Texas Southwestern Medical Center animal facility. All animal experiments were performed with the approval of UT Southwestern Committee on Animal Care.

### Expression of IGF-2-hFc

Originally IGF2-hFc was constructed by fusing IGF-2 and human IgG1 Fc under the control of CMV promoter [21]. The QuikChange Mutagenesis kit (Stratagene) was used to substitute Arg54-Arg55 with Ala-Leu to produce Arg-IGF2-hFc. The plasmid was transfected into 293T cells using Lipofectamine 2000 (Invitrogen). Conditioned medium was collected 48 hours after transfection. The conditioned medium with about 1 μg/ml of Arg-IGF-2-hFc as determined by western blotting was used in the subsequent staining of fetal liver or bone marrow cells.

### Flow cytometry, immunohistochemistry, and cytospin

Flow cytometry, immunohistochemistry, and cytospin were performed as we described previously [35,36].

Lin^−^ Sca-1^+^Kit^+^ cells were detected or sorted as described [37-39]. Ala-IGF2-hFc^+^ and Ala-IGF2-hFc^−^ cells were stained and sorted using a protocol similar to that described previously [21]. Briefly, 10^7^ cells were resuspended in 1 ml of conditioned medium (with ~1 μg/ml Arg-IGF2-hFc as determined by western blotting) at 4 ^o^C for 30 minutes followed by staining with anti-human IgG1 Fc-PE (Jackson ImmunoResearch). The conditioned medium from mock transfected cells was used as a control. When necessary, the co-staining of biotinylated Lin^+^ antibody cocktail followed by streptavidin-APC and anti-Sca-1-FITC was performed before sorting Lin^−^Sca-1^+^IGF2-hFc^+^ and Lin^−^Sca-1^+^IGF2-hFc^−^ cells in a FACSAria sorter.

For reconstitution analyses, peripheral blood cells were collected by retro-orbital bleeding. Red blood cells were lysed and stained with anti-CD45.2-FITC, anti-CD45.1-PE, anti-Thy1.2-PE, anti-B220-PE, anti-Mac1-PE, anti-Gr1-PE, or anti-Ter119-PE monoclonal antibodies (BD Pharmingen) as we described [39,40]. FACS analyses were performed on a FACSCalibur® instrument.

For flow cytometry analysis of leukemia cells, peripheral blood or bone marrow cells were stained with anti-Lineage-Biotin (followed by streptavidin-APC), anti-Mac1-APC, anti-Gr1-PE, anti-CD3-APC, anti-B220-PE, or anti-Kit-PE monoclonal antibodies (BD Pharmingen). Cell cycle status was determined by propidium iodide staining. For analysis of apoptosis, cells were stained with PE-conjugated anti-annexin V and 7-AAD (BD Pharmingen) according to the manufacturer’s instructions.

### Competitive reconstitution analysis

Indicated numbers of CD45.2 donor cells were mixed with 1 × 10^5^ freshly isolated CD45.1 competitor bone marrow cells, which were then injected intravenously via the retro-orbital route into CD45.1 mice previously irradiated with a total dose of 10 Gy. To measure reconstitution of transplanted mice, peripheral blood was collected by retro-orbital bleeding at the indicated times post-transplant and the presence of CD45.1^+^ and CD45.2^+^ cells in lymphoid and myeloid compartments were measured as we described [39].

### CML mouse model

The retrovirus transplantation CML mouse model was prepared essentially as described [25,26]. Bone marrow cells from 5-FU treated C57BL/6 mice or C57BL/6 fetal liver Lin^−^ cells were infected with retrovirus carrying MSCV-BCR/ABL-IRES-GFP. The transduced cells (5 × 10^5^) were transplanted intravenously into lethally irradiated (10 Gy) C57BL/6 recipients.

### Colony forming unit (CFU) assays

Cells from leukemia mice were plated in methylcellulose (M3534, Stem Cell Technologies) for CFU-GM assays according to the manufacturer’s protocols and our previously published protocol [35,37,39]. After 7 days, 2000 cells from three dishes were used for secondary replating.

### Western blotting

Cell lysates (100 μg samples) were separated by electrophoresis on a 4-12% SDS-polyacrylamide gel, and the proteins were electroblotted onto a nitrocellulose membrane. The membrane was probed with primary antibody for 1 hour at room temperature and then incubated with horseradish peroxidase-conjugated secondary antibody, which was detected with the chemiluminescence SuperSignal kit (Pierce).

### Quantitative RT-PCR

Total RNA was isolated from FACS-collected cells. First-strand cDNA was synthesized using SuperScript II RT (Invitrogen). Samples were analyzed in triplicate 25-μl reactions (300 nM each primer, 12.5 μl of Master mix) as adapted from the standard protocol provided in SYBR Green PCR Master Mix and RT-PCR Protocols provided by Applied Biosystems. Primers were purchased from Qiagen or Sigma. The default PCR protocol was used on an Applied Biosystems Prism 7000 Sequence Detection System. The mRNA level in each population was normalized to the level of *β-actin* RNA transcripts present in the same sample as described previously [41].

### Statistical analyses

Data are expressed as means ± SEM. Data were analyzed by Student’s *t* test and were considered statistically significant if *p* < 0.05. The survival rates of the two groups were analyzed using a log-rank test.

## Abbreviations

ALL: Acute lymphoblastic leukemia
AML: Acute myeloid leukemia
CML: Chronic myeloid leukemia
CML-ICs: Chronic myeloid leukemia initiating cells
HSCs: Hematopoietic stem cells
IGF: Insulin-like growth factor
IGF-IR: IGF type 1 receptor
IGF-IIR: IGF type 2 receptor
Ph: Philadelphia chromosome
TKI: Tyrosine kinase inhibitor
WT: Wild-type
